# Coverage of antenatal iron-folic acid and calcium distribution during pregnancy and their contextual determinants in the northeastern region of India

**DOI:** 10.3389/fnut.2022.894245

**Published:** 2022-07-18

**Authors:** Kaustubh Bora, Bhupen Barman, Star Pala, Ananya Das, Goter Doke, Amar Tripura

**Affiliations:** ^1^Hematology Division, ICMR-Regional Medical Research Centre North East Region, Dibrugarh, India; ^2^Department of General Medicine, North Eastern Indira Gandhi Regional Institute of Health and Medical Sciences, Shillong, India; ^3^Department of Community Medicine, North Eastern Indira Gandhi Regional Institute of Health and Medical Sciences, Shillong, India; ^4^Department of Obstetrics and Gynecology, North Eastern Indira Gandhi Regional Institute of Health and Medical Sciences, Shillong, India; ^5^Department of Obstetrics and Gynecology, Tomo Riba Institute of Health and Medical Sciences, Naharlagun, India; ^6^Department of Community Medicine, Agartala Government Medical College, Agartala, India

**Keywords:** pregnancy, micronutrient, supplement, anemia, preeclampsia, iron, folic acid, calcium

## Abstract

Iron-folic acid (IFA) and calcium supplementation are nutritional interventions recommended prophylactically (against maternal anemia and preeclampsia, respectively) to all antenatal mothers in India under basic antenatal care (ANC) services. Using Health Management Information System data (reporting period: 2018–19 to 2020–21), we mapped the coverage of antenatal IFA and calcium distribution across the remote northeastern region of India relative to the number of pregnant women (PW) who registered for ANC, disaggregated by states and districts. Variations in coverage were also investigated by subgroups based on contextual attributes, *viz*., physiography (hilly/ plateau/ plain), socioeconomic development (“aspirational”/ “non-aspirational”) and proportion of early ANC visits (low/ medium/ high). Full course of antenatal IFA and calcium supplements were received by 79.36 (95% CI: 79.31–79.40) and 61.26 (95% CI: 61.21–61.32) PW per 100 ANC registered women, respectively. There was widespread heterogeneity in outreach, with calcium coverage generally trailing behind IFA coverage. Among states, coverage of the two interventions (per 100 ANC registered women) was highest in Assam (97.06 and 78.11 PW, respectively) and lowest in Nagaland (24.87 and 16.77 PW, respectively). At the district-level, the two interventions failed to reach even 50 PW per 100 ANC registered women in 32 (out of 115) districts. The coverage tended to be inferior in districts that were hilly, “non-aspirational” and had low proportion of early ANC visits. The granular information provided by our findings will facilitate monitoring, root cause analyses, microplanning, informed resource allocation and tailoring of locally appropriate solutions to achieve targeted coverage improvements.

## Introduction

Pregnancy is a crucial juncture in a woman's reproductive life. Nutritional inadequacy during pregnancy may lead to adverse maternal and child health outcomes (1–5), with detrimental consequences even in later life (5–7). The World Health Organization (WHO) underscores the importance of good maternal nutrition for a positive pregnancy experience. Apart from encouraging nutritional education and healthy eating behavior during pregnancy, the WHO specifically advocates for supplementation of certain micronutrients within the ambit of basic antenatal care (ANC) services (8). That includes routine supplementation of iron-folic acid (IFA) and calcium (in populations with low dietary calcium intakes) as prophylaxis against maternal anemia and pre-eclampsia, respectively (8–10), which are important (but preventable) conditions responsible for considerable morbidity and mortality (8, 11–14). Promotion of maternal nutrition also resonates with the Sustainable Development Goals and the Decade of Action on Nutrition (2016–2025) proclaimed by United Nations (15–17).

In alignment with WHO guidelines, prophylactic IFA and calcium supplementation are recommended for all mothers in India as a part of the basic ANC package under National Health Mission (NHM). Accordingly, distribution of IFA (total 180 tablets, each containing 60 mg elemental iron and 500 μg folic acid) and calcium supplements (total 360 tablets, each containing 500 mg elemental calcium and 250 IU vitamin D_3_) are mandated in all pregnant Indian women for daily consumption (one IFA tablet and two calcium tablets) starting from second trimester and continuation for minimum 180 days during gestation (18, 19).

The northeastern region (NER) of India comprises of eight remote states (namely Arunachal Pradesh, Assam, Manipur, Meghalaya, Mizoram, Nagaland, Sikkim, and Tripura) that share a long international border (>5,100 km, i.e., nearly 99% of the region's boundary) with five neighboring countries. Located strategically between South Asia and South-east Asia, the region is geographically connected to the rest of India by a thin strip of land measuring ~22 kilometers at the narrowest section. It is inhabited by a sizeable tribal population and has limited industrialization with infrastructural/logistical challenges, as compared to other parts of the country (20). The NER states have a substantial burden of maternal and child health problems including malnutrition and micronutrient deficiencies (21–23). Despite appreciable strides and an epidemiological transition in India over the years, reproductive care and maternal/child health indicators in NER continue to be poor (23–28).

Thus, we evaluated the coverage of antenatal IFA and calcium distribution in NER across two important administrative level units, namely, states and districts. Additionally, contextual determinants that may impact the coverage were investigated.

## Methods

### Data source

We used Health Management Information System (HMIS) data for this study. The HMIS is a Government of India initiative that helps in monitoring NHM and other health programmes. It may be harnessed for programme evaluation, gap analysis, epidemiological insights, and health systems research (28–32). The HMIS network compiles monthly information regarding various health indicators (such as patient services, ANC, family planning, immunization, etc.) across India in the form of count data under specified headings/sub-headings (called “data elements”). This data is collected from health facilities all over the country and aggregated in a bottom-up manner (i.e., from Facility level to Sub-district, District, State and National levels) through a web-based computerized interface, using standardized and uniform data entry formats (28). The information is verified for accuracy and completeness using quality-check mechanisms. The curated data is then hosted in the public domain every financial year (i.e., April to March cycle).

### Data definitions and retrieval

The HMIS records antenatal information under the heading “*Ante Natal Care (ANC)*.” From 2017–18 onwards, the data elements “*Number of PW given 180 Iron Folic Acid (IFA) tablets*” and “*Number of PW given 360 Calcium tablets*” (PW in HMIS indicates pregnant women) were included under this heading. We used these data elements (as numerators) to define the coverage of antenatal IFA and calcium distribution between April 2018 and March 2021 (i.e., 2018–19, 2019–20, and 2020–21 reporting periods), relative to the number of pregnant women who had registered for ANC (as denominator). The denominator information was available from the data element “*Total number of PW registered for ANC*”.

The above data pertaining to NER were retrieved in a state- and district-wise manner from HMIS portal (https://hmis.nhp.gov.in/#!/). It included all the eight NER states (Supplementary Figure 1A). We also retrieved overall data for India (encompassing all states/union territories) to compare how the NER coverage fared vis-à-vis the national coverage. In recent times, the NER districts were reorganized extensively (however, state boundaries were unaffected). Therefore, assessment at district-level was limited to the most recent reporting year (i.e., 2020–21) which included information from 120 districts (Supplementary Figure 1B). Of these, two new districts (namely, Jiribam and Noklak) were yet to start reporting antenatal IFA and calcium data, while data from three new districts (namely, South Salmara-Mancachar, Lower Siang and Pherzawl) revealed outliers. These five districts were excluded from the district-level analysis (Supplementary Figure 1C).

### Contextual variables of interest and subgroups

Situated in the Eastern Himalayan region, NER has a challenging terrain that is hilly and rocky interspersed with alluvial plains and valleys. Broadly, three physiographical features are identifiable in NER, *viz*., hills/mountains (spanning Sikkim Himalayas, Arunachal Himalayas, Patkai Hills, Naga Hills, Barail range, Lushai Hills, and hill ranges of Manipur and Tripura), plateau (formed by Karbi-Meghalaya plateau), and plains (including Brahmaputra and Barak plains of Assam, Imphal valley of Manipur, and plains of Tripura) (33, 34). We explored if antenatal IFA and calcium coverage in NER varied according to this physiographical classification that yielded 65 predominantly hilly/mountainous districts, 13 plateau districts, and 37 plain districts (Supplementary Figure 1D).

The coverage of antenatal IFA and calcium distribution was also assessed in relation to socioeconomic development. In January 2018, the Government of India identified the country's most underdeveloped districts that needed immediate and concerted improvements. These districts, identified through comprehensive evaluation of 49 key performance indicators across five broad socioeconomic themes (i.e., Health and Nutrition, Education, Agriculture and Water Resources, Financial Inclusion and Skill Development, and Infrastructure), were termed as “aspirational districts” (35). Fourteen such aspirational districts were identified in NER–seven districts in Assam (Baksa, Barpeta, Darrang, Dhubri, Goalpara, Hailakandi, Udalguri), and one district each in Arunachal Pradesh (Namsai), Manipur (Chandel), Meghalaya (Ri-bhoi), Mizoram (Mamit), Nagaland (Kiphire), Sikkim (West Sikkim) and Tripura (Dhalai) (35). The IFA and calcium distribution in these 14 districts were compared with that of the “non-aspirational” or “other” NER districts.

The WHO recommends “early ANC visit,” entailing ANC initiation in all pregnant women within first trimester, i.e., gestational age of <12 weeks (8). Early ANC brings the pregnant mother under the fold of ANC by introducing her to various healthcare services recommended at different stages of pregnancy. The fifth round of the National Family Health Survey (NFHS-5), conducted recently in 103 NER districts, found that the proportion of early ANC visits (median: 59.4%) ranged from 27.1% (in Tuensang) to 90.6% (in Thoubal) (27). Therefore, to investigate if antenatal IFA and calcium coverage varied according to the proportion of mothers undergoing early ANC visits, NER districts were stratified using tertile-based cut-offs as: districts with low (i.e., 27.1 to 54.8%), medium (i.e., 54.9 to 65.8%), and high (i.e., 65.9 to 90.6%) proportion of mothers undergoing early ANC visits.

### Statistical analysis

The coverage of antenatal IFA and calcium distribution in NER states was expressed in terms of number of pregnant women who were given full courses of these supplements (i.e., 180 tablets and 360 tablets, respectively) per 100 ANC registered women. The accompanying 95% confidence intervals (CIs) were calculated by Wilson score method. District-wise coverage was illustrated through choropleth maps. Coverage variations were summarized using coefficient of variation (CV), calculated as ratio of standard deviation to arithmetic mean in percentage. This was computed for the eight states (inter-state CV) and 115 districts (inter-district CV). Variations in coverage among districts within a state were also analyzed (using intrastate CV) and graphically depicted (using boxplots). The contextual factors influencing antenatal IFA and calcium distribution were probed by subgroup analysis (described above). The coverage achieved in subgroups under a particular grouping were considered to be significantly different if the associated 95% CIs were non-overlapping. The magnitude of such differences was quantified using relative coverage difference (CD) in percentage terms and coverage ratio (CR), with the subgroup exhibiting the highest coverage (within that grouping) serving as reference.

Multivariate regression was performed to substantiate if contextual factors had significant effects on the district-level IFA and calcium coverage values (outcome variable). Accordingly, physiographical category (hilly/ plateau/ plain), proportion of early ANC visits (in a continuous scale), and socioeconomic development category (“aspirational”/ “non-aspirational”) of the NER districts were included as explanatory variables. A two-sided *P* < 0.05 was applied as threshold for statistical significance.

We used Microsoft Excel (Microsoft Office Professional Plus 2019, Microsoft Corp.) and JASP 0.15.0 (JASP, University of Amsterdam) software suites for data analyses.

## Results

According to HMIS data from NER collected during the reporting period (i.e., between April 2018 and March 2021), the full courses of antenatal IFA and calcium supplements were provided to 2,457,883 and 1,897,418 pregnant mothers, respectively. Altogether 3,097,274 women in the region had registered for ANC in that period.

### Antenatal distribution of IFA and calcium supplements in NER states

The coverage of antenatal IFA distribution in NER states during the 2018–21 reporting period was 79.36 (95% CI: 79.31–79.40) pregnant women per 100 ANC registered women (Table 1A), and that for calcium was 61.26 (95% CI: 61.21–61.32) pregnant women per 100 ANC registered women (Table 1B). The national coverage during that period was relatively better, at 88.13 (95% CI: 88.12–88.13) and 69.86 (95% CI: 69.85–69.87) pregnant women per 100 ANC registered women, respectively. The outreach of antenatal IFA and calcium distribution among the individual NER states were highest in Assam, benefitting 97.06 and 78.11 pregnant women per 100 ANC registered women, respectively. By contrast, coverage of the two interventions were lowest in Nagaland, reaching only 24.87 and 16.77 pregnant women per 100 ANC registered women, respectively.

**Table 1A:** Coverage of iron-folic acid tablet distribution among pregnant women in the states of northeast India.

**State/** **Region**	**Pregnant women who received full course of 180 iron-folic acid tablets per 100 ANC registered women**			
	**(95% CI)**			
	**2018-19**	**2019-20**	**2020-21**	**All 3 years combined**
India (national level)	84.78 (84.77–84.79)	89.22 (89.21–89.23)	90.47 (90.46–90.48)	88.13 (88.12–88.13)
All northeastern states	77.32 (77.26–77.40)	79.70 (79.62–79.78)	81.23 (81.15–81.30)	79.36 (79.31–79.40)
Arunachal Pradesh	44.87 (44.33–45.42)	58.11 (57.55–58.66)	54.33 (53.78–54.88)	52.31 (51.99–52.63)
Assam	97.57 (97.53–97.60)	98.34 (98.31–98.37)	95.20 (95.15–95.25)	97.06 (97.04–97.09)
Manipur	38.77 (38.37–39.17)	49.30 (48.88–49.72)	42.62 (42.17–43.08)	43.56 (43.32–43.81)
Meghalaya	33.50 (33.25–33.75)	42.60 (42.34–42.86)	58.55 (58.26–58.84)	43.84 (43.68–43.99)
Mizoram	39.42 (38.79–40.06)	50.72 (50.08–51.36)	74.30 (73.72–74.86)	54.76 (54.39–55.13)
Nagaland	15.57 (15.21–15.94)	22.98 (22.57–23.40)	37.68 (37.16–38.20)	24.87 (24.62–25.13)
Sikkim	69.68 (68.75–70.60)	63.79 (62.81–64.76)	81.45 (80.57–82.29)	71.10 (70.55–71.64)
Tripura	56.49 (56.13–56.86)	46.75 (46.37–47.13)	42.33 (41.95–42.71)	48.83 (48.61–49.05)

*ANC, antenatal care; CI, confidence interval*.

**Table 1B:** Coverage of iron-folic acid tablet distribution among pregnant women in the states of northeast India.

**State/** **Region**	**Pregnant women who received full course of 360 calcium tablets per 100 ANC registered women (95% CI)**			
	**2018-19**	**2019-20**	**2020-21**	**All 3 years combined**
India (national level)	57.28 (57.27–57.30)	72.02 (72.06–72.09)	80.63 (80.62–80.65)	69.86 (69.85–69.87)
All northeastern states	46.31 (46.22–46.41)	71.29 (71.20–71.37)	66.94 (66.84–67.03)	61.26 (61.21–61.32)
Arunachal Pradesh	29.21 (28.72–29.71)	52.59 (52.02–53.15)	29.51 (29.01–30.02)	36.82 (36.51–37.13)
Assam	60.38 (60.27–60.50)	90.79 (90.72–90.86)	83.79 (83.70–83.88)	78.11 (78.06–78.17)
Manipur	19.79 (19.47–20.12)	21.62 (21.28–21.97)	27.52 (27.11–27.93)	22.66 (22.45–22.87)
Meghalaya	22.32 (22.10–22.54)	37.41 (37.16–37.67)	31.39 (31.12–31.66)	30.26 (30.12–30.40)
Mizoram	20.05 (19.53–20.57)	43.81 (43.17–44.44)	48.78 (48.13–49.43)	37.62 (37.26–37.99)
Nagaland	6.44 (6.20–6.69)	15.65 (15.29–16.01)	29.82 (29.33–30.32)	16.77 (16.55–16.99)
Sikkim	61.93 (60.49–62.45)	66.39 (65.43–67.34)	81.50 (80.63–82.34)	69.28 (68.73–69.83)
Tripura	12.22 (11.98–12.46)	34.69 (34.33–35.05)	25.39 (25.06–25.73)	23.78 (23.59–23.96)

*ANC, antenatal care; CI, confidence interval*.

During individual years of the 2018–21 reporting period, antenatal IFA distribution in NER increased from 77.32 (in 2018–19) to 79.70 (in 2019–20) to 81.23 (in 2020–21) pregnant women, per 100 ANC registered women (Table 1A). However, antenatal calcium distribution witnessed a spike from 46.31 (in 2018–19) to 71.29 (in 2019–20) followed by a decline to 66.94 (in 2020–21) pregnant women, per 100 ANC registered women (Table 1B). Among various NER states, only Mizoram, Nagaland and Sikkim witnessed continuous yearly increments in IFA and calcium coverage rates.

Overall, calcium distribution in NER states usually lagged behind IFA distribution, except in the state of Sikkim during 2019–20 (when calcium coverage was better than IFA coverage) and 2020–21 (when IFA and calcium coverage were comparable).

### Antenatal distribution of IFA and calcium supplements in NER districts

**Figure 1:**
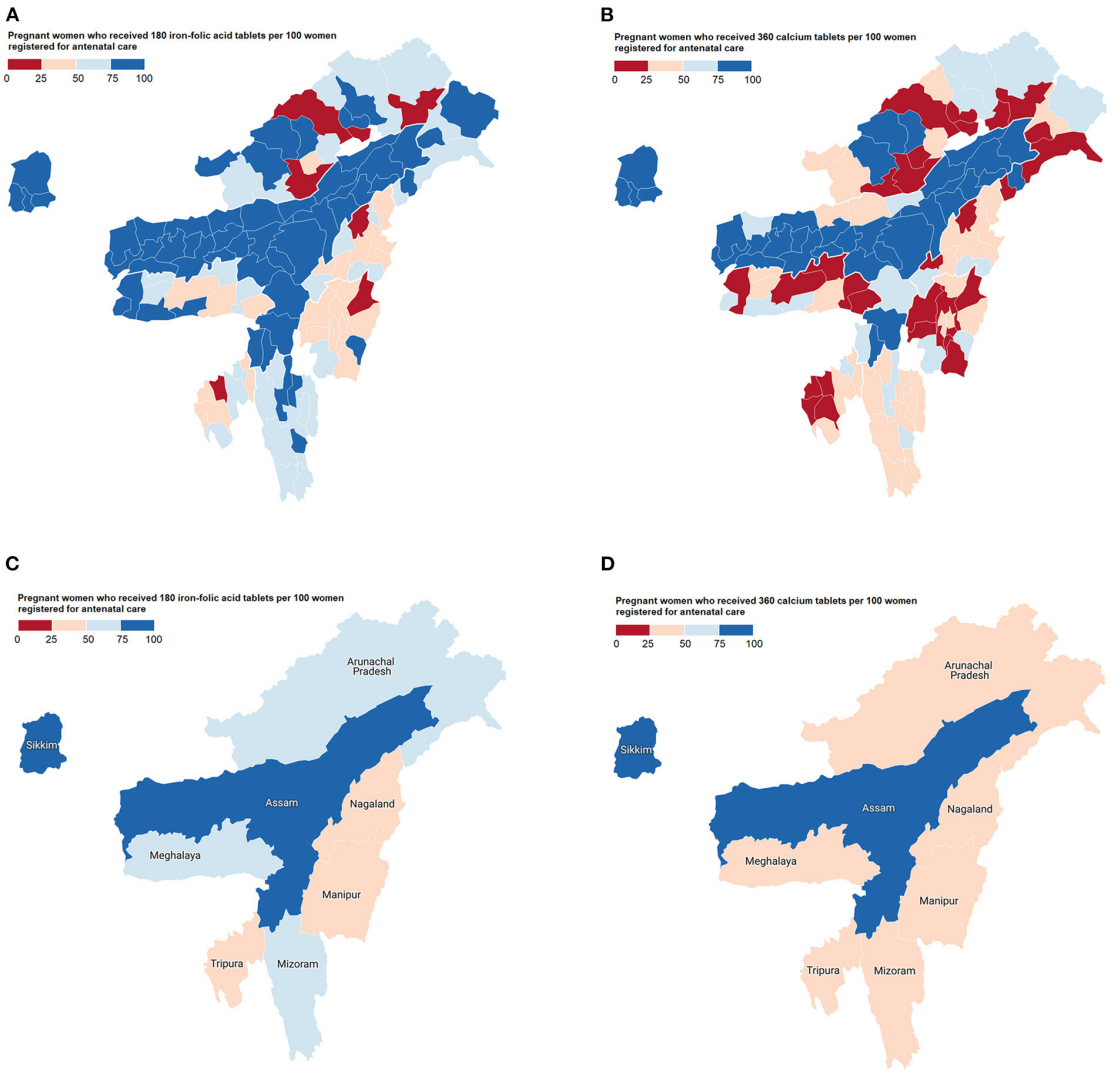
Choropleth maps showing the distribution of antenatal micronutrient supplements across northeast India in 2020–21. **(A)** Iron-folic acid coverage by districts; **(B)** Calcium coverage by districts; **(C)** Iron-folic acid coverage by states; and **(D)** Calcium coverage by states.

Figures 1A,B highlight IFA and calcium distribution among antenatal mothers from NER by districts during 2020–21 (corresponding state-level distribution shown in Figures 1C,D). The IFA coverage ranged from 8.74 (Ukhrul district, Manipur) to 99.94 (Karimganj district, Assam) pregnant women per 100 ANC registered women (Figure 1A). The calcium coverage ranged from 2.65 (Kakching district, Manipur) to 99.85 (East Kameng district, Arunachal Pradesh) pregnant women per 100 ANC registered women (Figure 1B). In 32 out of 115 districts (*i.e*., 27.8% districts in NER), antenatal IFA was distributed to <50 pregnant women per 100 ANC registered women. With respect to calcium, there were 63 such poor coverage districts (*i.e*., 54.8% districts in the region). The situation was particularly deplorable in seven districts, where IFA and calcium coverage reached <25 pregnant women per 100 ANC registered women. This included four districts from Arunachal Pradesh (Lower Dibang valley, Upper Subanshiri, Lepa Rada, Papum Pare) and one district each from Manipur (Ukhrul), Nagaland (Mokokchung) and Tripura (Khowai).

### Variations in antenatal IFA and calcium distribution across NER

Antenatal IFA and calcium distribution were quite variable across NER. The coverage variations among states (inter-state CV) amounted to 34.4% (for IFA) and 54.8% (for calcium) in 2020–21. Among districts (inter-district CV), the variations amounted to 36.5% and 57.1%, respectively. Variations were observed even among districts from the same state (Supplementary Figures 2A,B). Sikkim, with the least intrastate CV values (IFA 4.5%, calcium 4.9%), was the only NER state where outreach of both these interventions in all constituent districts exceeded 75 pregnant women per 100 ANC registered women (Table 2). High IFA coverage, exceeding 75 pregnant women per 100 ANC registered women, was also recorded in all districts of Assam (intrastate CV 5.0%).

**Table 2:** Variations in antenatal iron-folic acid and calcium coverage distribution among districts belonging to different states of northeast India, 2020–21.

**States in northeast India**	**No. of districts**	**Pregnant women who received full course of iron-folic acid supplements per 100 ANC registered women**			**Pregnant women who received full course of calcium supplements per 100 ANC registered women**		
		**Median coverage (range)**	**Mean coverage (SD)**	**Intrastate CV**	**Median coverage (range)**	**Mean coverage (SD)**	**Intrastate CV**
Arunachal Pradesh	24	65.0 (16.6–99.8)	64.6 (24.7)	38.2%	31.5 (8.3–99.8)	40.9 (28.7)	70.1%
Assam	32	95.8 (84.7–99.9)	94.7 (4.7)	5.0%	86.5 (30.2–97.0)	81.5 (15.9)	19.5%
Manipur	14	43.8 (8.7–91.0)	43.6 (18.0)	41.3%	21.3 (2.7–63.6)	25.0 (17.3)	68.9%
Meghalaya	11	66.8 (44.2–86.5)	64.8 (16.5)	25.4%	33.0 (12.9–58.2)	35.2 (15.7)	44.4%
Mizoram	11	68.2 (54.8–95.6)	70.9 (13.6)	19.2%	39.0 (27.7–71.3)	44.6 (14.0)	31.5%
Nagaland	11	41.9 (20.7–68.4)	44.0 (13.3)	30.3%	37.4 (17.6–66.1)	37.4 (13.6)	36.5%
Sikkim	4	84.0 (77.9–86.2)	83.0 (3.7)	4.5%	85.2 (77.1–85.4)	83.2 (4.1)	4.9%
Tripura	8	38.8 (24.9–64.3)	43.7 (15.3)	35.0%	25.1 (10.9–52.2)	27.4 (15.6)	57.0%

*ANC, antenatal care; SD, standard deviation; CV, co-efficient of variation*.

### Antenatal IFA and calcium distribution in NER by contextual factors

Subgroup analysis revealed differential patterns in the antenatal distribution of IFA and calcium supplements across NER by several contextual factors (Tables 3A,B). The predominantly hilly/mountainous and plateau districts lagged behind the plain districts (reference) in IFA and calcium distribution. Pregnant mothers from such districts were 0.57 and 0.72 times less likely (i.e., 42.54% and 27.74% lesser coverage) to receive IFA supplements, and 0.46 and 0.52 times less likely (i.e., 53.72% and 47.60% lesser coverage) to receive calcium supplements, respectively than their counterparts in plain districts. With respect to socioeconomic development, IFA and calcium distribution were superior in aspirational districts (by 14.09 and 22.01%, respectively) as compared to “other” districts. Besides, a graded difference in calcium (but not IFA) distribution was observed according to the proportion of mothers who underwent early ANC visits. Thus, districts where the proportion of mothers undergoing early ANC visits was low and medium trailed in calcium distribution (by 13.19 and 6.48%, respectively) behind districts with a high proportion of early ANC visits (reference).

**Table 3A:** Differential patterns in antenatal iron-folic acid coverage across districts of northeast India by contextual factors, 2020–21.

**District subgroups by contextual factors**	**Pregnant women who received**		
	**full course of iron-folic acid**		
	**supplements per 100 ANC**		
	**registered women**		
	**Coverage (95% CI)**	**Relative CD**	**CR**
**Physiographical grouping**			
Hilly/mountainous	52.04 (51.79–52.29)	−42.54%	0.57
Plateau	65.45 (65.19–66.70)	−27.74%	0.72
Plain	90.57 (90.50–90.64)	Reference	Reference
**Special categories (under Transformation of Aspirational Districts initiative)**			
Aspirational districts	90.98 (90.86–91.10)	Reference	Reference
Other districts	78.16 (78.07–78.26)	−14.09%	0.85
**Grouping based on proportion of mothers who had early ANC**			
Low (27.1 to 54.8%)	81.51 (81.36–81.65)	Reference	Reference
Medium (54.9 to 65.8%)	81.21 (81.07–81.34)	−0.40%	1.00
High (65.9 to 90.6%)	81.35 (81.22–81.49)	−0.20%	1.00

*ANC, antenatal care; CI, confidence interval; CD, coverage difference; CR, coverage ratio*.

*While comparing the coverage across different sub-groups within a grouping, the sub-group with the highest coverage was treated as reference*.

**Table 3B:** Differential patterns in antenatal calcium coverage across districts of northeast India by contextual factors, 2020–21.

**District subgroups by contextual factors**	**Pregnant women who received**		
	**full course of calcium**		
	**supplements per 100 ANC**		
	**registered women**		
	**Coverage (95% CI)**	**Relative CD**	**CR**
**Physiographical grouping**			
Hilly/mountainous	36.28 (36.04–36.52)	−53.72%	0.46
Plateau	41.08 (40.81–41.34)	−47.60%	0.52
Plain	78.40 (78.30–78.49)	Reference	Reference
**Special categories (under Transformation of Aspirational Districts initiative)**			
Aspirational districts	80.36 (80.19–80.53)	Reference	Reference
Other districts	62.67 (62.56–62.78)	−22.01%	0.78
**Grouping based on proportion of mothers who had early ANC**			
Low (27.1 to 54.8%)	62.01 (61.83–62.19)	−13.19%	0.87
Medium (54.9 to 65.8%)	66.80 (66.65–66.96)	−6.48%	0.94
High (65.9 to 90.6%)	71.43 (71.28–71.59)	Reference	Reference

*ANC, antenatal care; CI, confidence interval; CD, coverage difference; CR, coverage ratio*.

*While comparing the coverage across different sub-groups within a grouping, the sub-group with the highest coverage was treated as reference*.

Regression models indicated that the examined contextual factors collectively explained 20.2% and 18.8% of the variances in IFA (adjusted *R*^2^ = 0.202, *F*-value = 7.47, *P* < 0.001) and calcium (adjusted *R*^2^ = 0.188, *F*-value = 6.92, *P* < 0.001) coverage, respectively (Supplementary Table 1). Of these, physiographical category (hilly districts) had the most profound association, affecting both IFA (β-coefficient = −23.92, *P* < 0.001) and calcium (β-coefficient = −27.59, *P* < 0.001) coverage negatively.

## Discussion

Antenatal supplementation of IFA and calcium are evidence-based nutritional interventions for reducing the risks of maternal anemia and pre-eclampsia, respectively (8–10). Anemia during pregnancy is an important public health problem in NER (27, 36), with prevalence between 22.2 and 61.5% across various states of the region (Supplementary Table 2). Although IFA supplementation is the most commonly employed intervention against anemia, all anemic cases are not IFA-corrigible and the relative benefits vary across settings (37–40). Nutritional supplements containing iron and folic acid were found to improve hemoglobin concentrations appreciably during a multicentric study in anemic women of reproductive age (WRA) from NER (38). In fact, the estimated odds of reducing anemia with every milligram increase in daily iron intake among WRA across India was observed to be the highest in NER states (particularly Manipur, Nagaland, Mizoram, Sikkim, and Arunachal Pradesh), ranging from 0.88 (95% CI: 0.84–0.91) to 0.96 (95% CI: 0.94–0.99). Of these, Manipur, Nagaland and Mizoram had relatively low anemia prevalence, but also low per capita daily iron intake (40). Reliable estimates from NER on the burden of folate deficiency during pregnancy are currently unavailable. However, surveys among WRA from different communities in NER have documented low intakes of dietary folate (41, 42). Interestingly, the recent Comprehensive National Nutritional Survey found preponderance of folate deficiency among adolescents from NER (43). The highest prevalence was observed in Nagaland (88.9%). The magnitude in Assam (73.3%), Meghalaya (61.5%) and Arunachal Pradesh (47.9%) also exceeded the national prevalence (36.7%). Similar patterns are plausible in antenatal mothers and should be explored in future. The NER states ranked among the country's highest in terms of pre-eclampsia burden, as well (44). The estimated pre-eclampsia prevalence in every NER state was higher than national average (Supplementary Table 2). This is further complicated by the fact that average daily calcium intake in NER states (particularly in Manipur, Nagaland, Meghalaya, Assam, Arunachal Pradesh, Mizoram, and Tripura) are among the country's lowest (45). Thus, antenatal IFA and calcium supplementation activities in NER are of special interest.

With this background, the present study describes the outreach of antenatal IFA and calcium distribution in pregnant women from northeast India, disaggregated by policy-relevant administrative units. The outreach of these interventions was uneven and exhibited variations across the region. Both demand-side (e.g., health-seeking behavior, health service utilization, etc.) and supply-side (e.g., health facilities, health worker motivation, supply chains and stocks, etc.) factors are critical for programme success (46, 47). The findings from our study provide valuable pointers about important contextual factors that may have contributed toward the coverage disparities.

The disparities were most prominent in relation to physiographical characteristics. While the plain districts were best performers in terms of coverage, the districts that were predominantly hilly/mountainous were the least covered followed by the plateau districts. These differential patterns were consistent for both IFA and calcium distribution. Geographical remoteness and difficult terrain are increasingly recognized as major impediments to healthcare services (47–49). Operating at the interface of supply-demand interactions, such challenges can affect both health delivery (on the part of healthcare providers) and health seeking (on the part of intended beneficiaries). By and large, the NER states are mostly rugged and mountainous (with the exception of Assam) and further have dense forest cover encompassing >70% of the geographical area (with the exception of Assam and Sikkim) (33, 50). Recently, the utilization of maternal and child health services in NER states (like Nagaland, Arunachal Pradesh and Sikkim) was reported to be surprisingly low despite presence of adequate health facilities due to accessibility problems in the complex terrain (32, 51). Moreover, the average distance to health facilities in many parts of NER (especially in Meghalaya and Mizoram) was found to be significantly higher than the national average (51). Such inconveniences may hamper outreach of the two interventions in the region.

On the other hand, coverage of IFA and calcium distribution was counterintuitively better in the aspirational districts as opposed to the “other” districts. The aspirational districts are otherwise considered to be the socioeconomically most underdeveloped. However, the special emphasis placed on the progress of aspirational districts in recent times may have contributed to the superior programme coverage in these districts. Under the “Transformation of Aspirational Districts” initiative launched in January 2018, these districts are regularly monitored and encouraged to develop and replicate practices for achieving overall transformation and improvement across socioeconomic paradigms to be at par with the “best” districts (35, 52).

The distribution of calcium supplements among pregnant mothers in NER also varied as a function of early ANC visits. Early ANC visit is an opportunity for healthcare providers to engage with pregnant mothers early on, and for sensitizing and encouraging them to avail various ANC services (including nutritional support) during the course of pregnancy for achieving positive outcomes (8, 53). It is plausible that mothers who underwent early ANC visits were more aware and better informed about antenatal calcium supplementation. This may have aided in greater programme utilization and coverage in the districts with higher proportion of early ANC visits.

Unlike calcium supplements however, the antenatal IFA distribution rates were high (and comparable) irrespective of the proportion of early ANC visits. It indicates existence of additional challenges that are specific for calcium supplementation. From programme point of view, IFA supplementation efforts for preventing maternal anemia in India date back to nearly half a century when the Nutritional Anemia Prophylaxis Programme was launched in 1970 (54). This has evolved over the years and is presently implemented through Intensified National Iron Plus Initiative (I-NIPI) of the Anemia Mukt Bharat strategy under which the current set of antenatal IFA supplementation guidelines have been issued (18). In contrast, antenatal calcium intervention (introduced in 2014) against pre-eclampsia is very recent (19). Thus, awareness about antenatal calcium supplementation (or pre-eclampsia) among the general public and/or health workers may be relatively low, which may have contributed toward its poorer coverage (compared to IFA). Additionally, there could be supply-side bottlenecks in the calcium distribution ecosystem, *viz*., stock unavailability, procurement problems, and difficulties in warehousing and timely delivery. While such disruptions are known to influence the effective coverage of public health nutritional programmes in general (46, 47, 55), we are unaware of studies that have investigated supply chain aspects of calcium supplements in particular. The fact that calcium coverage generally lagged behind IFA coverage in the NER states and district subgroups (in spite of similar operational guidelines) lends further credibility to such possibilities which are systemic in nature and perhaps specific for calcium supplements.

Mobile distribution posts and depots may facilitate coverage in remote and interior locations. Recently, drones were field-tested and deployed by Indian Council of Medical Research (ICMR) for delivering vaccines in hard-to-reach locations of NER (56). Following cost considerations and feasibility studies, such technological solutions may act as force-multipliers to plug coverage gaps in geographically challenging/inaccessible terrain (57). Simultaneously, measures for overcoming supply chain bottlenecks must be initiated, *viz*., timely and accurate forecasting of IFA and calcium supplement requirements, user-friendly and streamlined procurement processes, and creation of sufficient warehousing and logistical infrastructure for inventory management. These efforts should be complemented by sustained community-based awareness and behavioral change campaigns for improving the adherence and compliance to these antenatal nutritional support interventions, especially in locations having poor uptake of ANC services among the population.

We acknowledge the following limitations of the study. Firstly, with respect to the antenatal IFA and calcium distribution, the HMIS platform records only those antenatal mothers who received the recommended dosage of the supplements in entirety (i.e., full course of 180 IFA and 360 calcium tablets). Due to this “all or none” documentation, it was not possible to examine the phenomenon of partial or incomplete coverage of these programmes. Secondly, we could not assess the relationship of IFA and calcium coverage with the number of ANC visits as this information was not available. Thirdly, we could not assess if the mothers receiving the supplements had actually adhered to them as per recommended dosage and schedule. Coverage data can be more effective when informed by compliance/acceptance data. But this information was unavailable with HMIS. Fourthly, although the examined contextual factors were found to significantly influence antenatal IFA and calcium distribution in NER, they explained only 18–20% of the coverage variance. It points toward the role of other important determinants at play that need to be investigated. Particularly, the “immediate reasons” for the lapses in coverage (such as lack of supplies, conveyance issues, poor awareness/motivation, individual challenges, etc.) could not be ascertained. These limitations were beyond the scope of the present study and require future exploration with the help of suitably designed community-based studies. Our findings would serve as a valuable resource and starting point in this regard.

To conclude, this study provides an overview about the coverage of antenatal IFA and calcium distribution among pregnant women from the eight states of northeast India. The coverage values were highly heterogeneous, with a nearly four-fold difference between the highest coverage and lowest coverage states. Apart from the between state differences, there were variations in coverage even among districts within a state. Factors like physiography, socioeconomic conditions and early ANC visits in the districts emerged as potentially important contextual determinants. The sub-regional variations and disparities unraveled by our study warrant regular monitoring and further investigations to identify the “immediate reasons” that limited the outreach of these nutritional interventions. Insights from the present study will help in identifying the root causes responsible for coverage inadequacies and thereby to start planning actions for improving the situation. Successful implementation of these interventions would require targetted remedies and informed allocation of resources. In that context, granular and disaggregated “local” coverage scenario described at the level of states/districts by our study would be useful for carrying out root cause analyses, microplanning and tailoring of locally appropriate solutions to enhance programme coverage.

## Data availability statement

The datasets presented in this study can be found in online repositories. The names of the repository/repositories and accession number(s) can be found below: Health Management Information System (HMIS) datasets, accessible at: https://hmis.nhp.gov.in/#!/.

## Ethics statement

Ethical review and approval was not required for the study on human participants in accordance with the local legislation and institutional requirements. Written informed consent for participation was not required for this study in accordance with the national legislation and the institutional requirements.

## Author contributions

KB: conceptualization, data collection, data analysis, interpretation, and writing of the manuscript. BB: literature review and critical review of the manuscript. SP and AD: data interpretation and critical review of the manuscript. GD and AT: critical review of the manuscript. All authors contributed to the article and approved the final version.

## Conflict of interest

The authors declare that the research was conducted in the absence of any commercial or financial relationships that could be construed as a potential conflict of interest.

## Publisher's note

All claims expressed in this article are solely those of the authors and do not necessarily represent those of their affiliated organizations, or those of the publisher, the editors and the reviewers. Any product that may be evaluated in this article, or claim that may be made by its manufacturer, is not guaranteed or endorsed by the publisher.
