# Supplementary material for: Coverage of antenatal iron-folic acid and calcium distribution during pregnancy and their contextual determinants in the northeastern region of India
Source: Front Nutr. 2022 Jul 18;9:894245. doi: 10.3389/fnut.2022.894245 (PMC9339897; doi:10.3389/fnut.2022.894245)

Supplementary Figure 1. Maps showing the geographical distribution of the study. **(A)** Northeastern region (NER) of India; **(B)** States in the NER; **(C)** Districts in the NER (the five unshaded districts marked by arrowheads were excluded from the district-level analysis; details provided in methodology); and **(D)** Predominant physiographical feature of the NER districts.

(A)

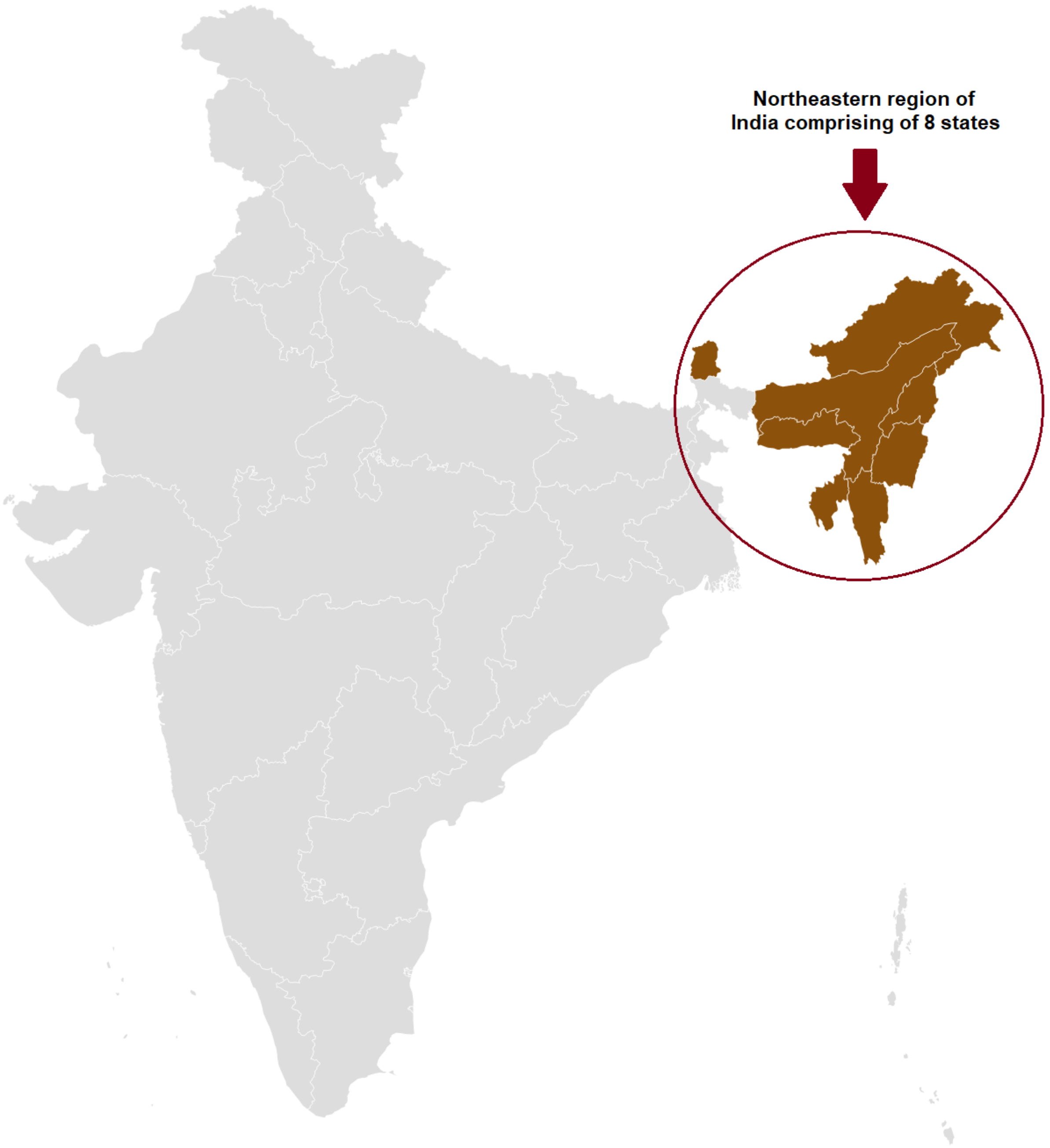

(B)

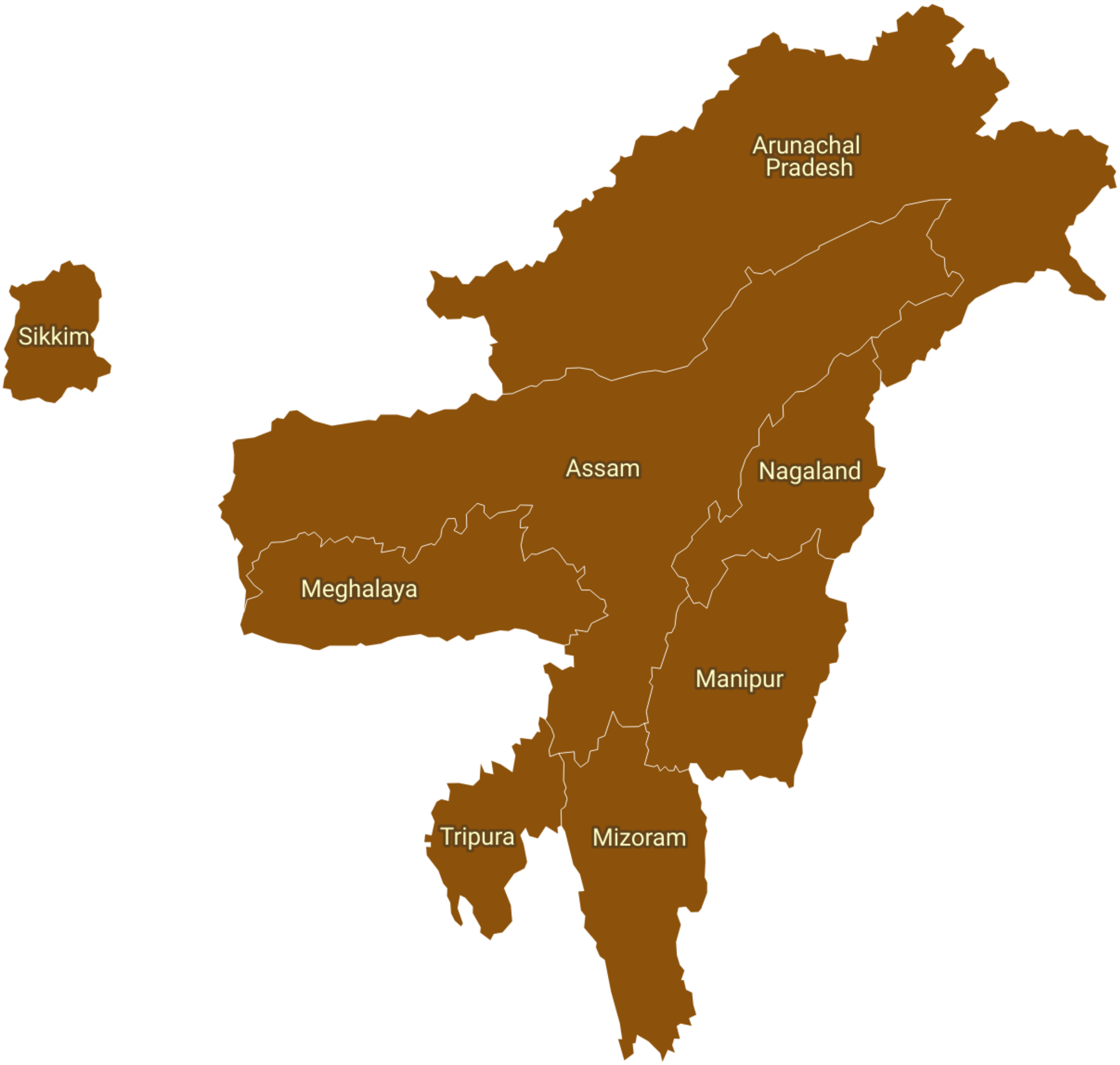
Arunachal Pradesh
Sikkim
Assam
Nagaland
Meghalaya
Manipur
Tripura
Mizoram

(C)

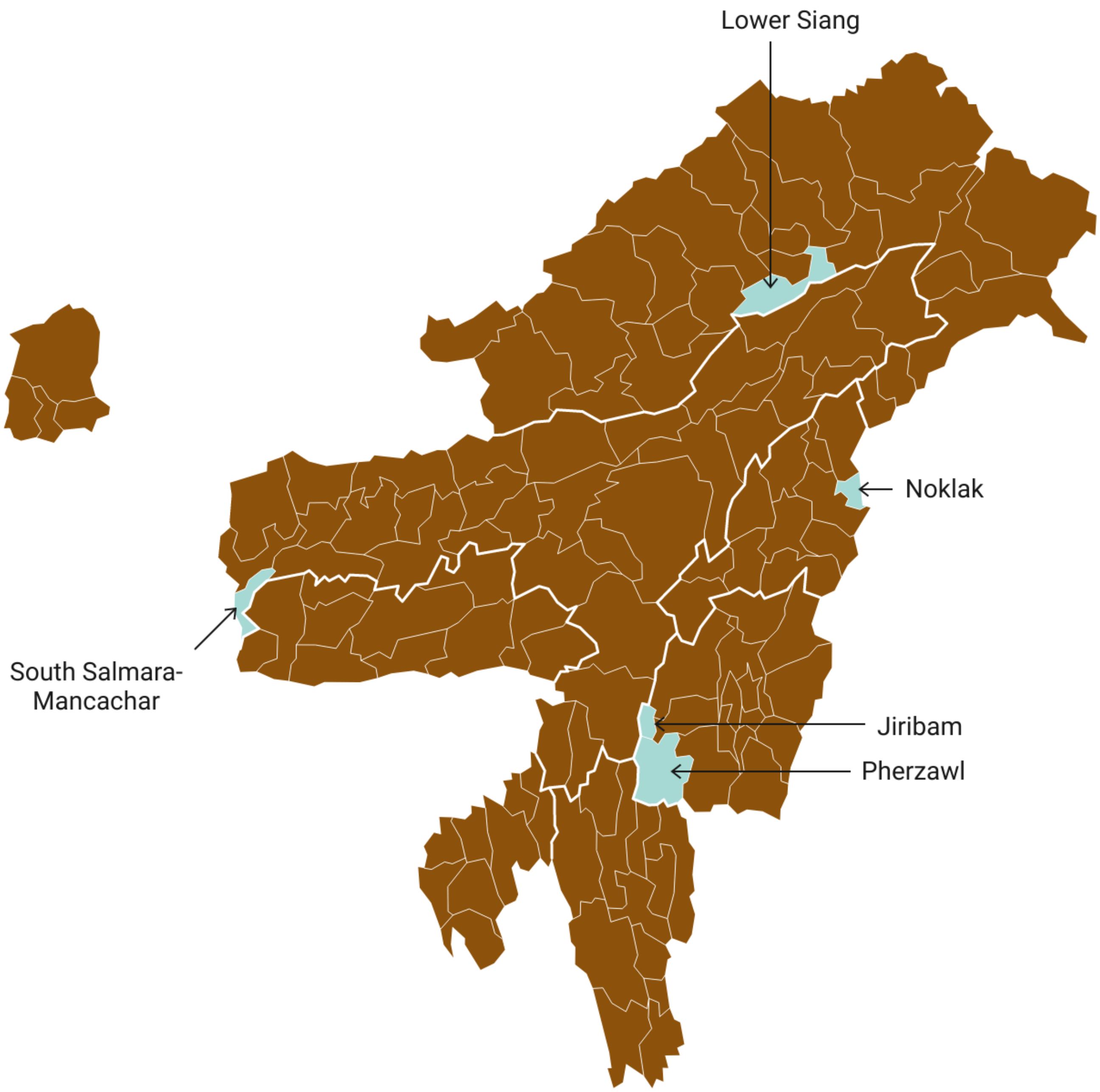
Lower Siang
Noklak
South Salmara-
Mancachar
Jiribam
Pherzawl

(D)

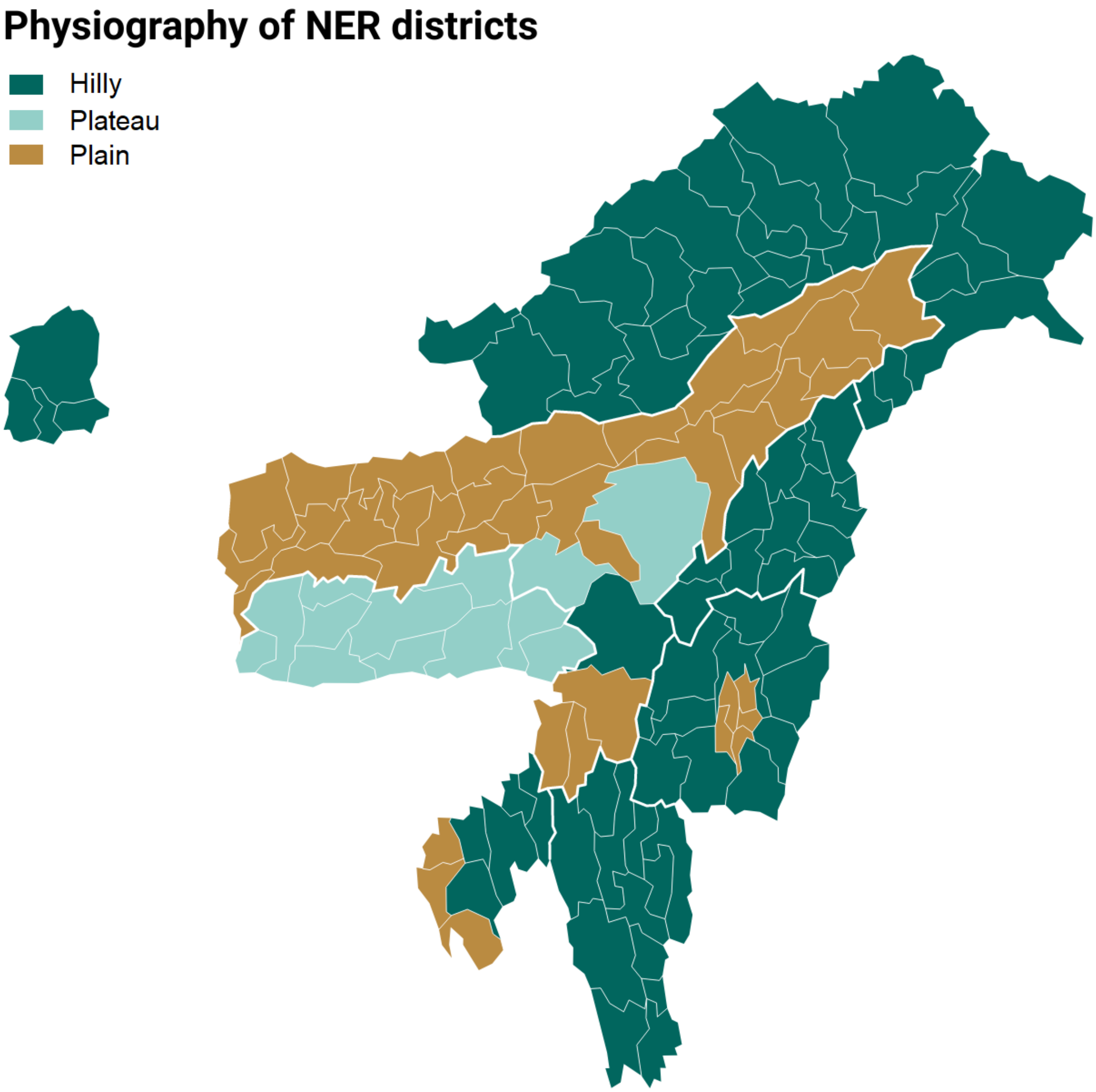

Supplement: Supplementary file 1 [file Data_Sheet_1.PDF]
