# Supplementary material for: Coverage of antenatal iron-folic acid and calcium distribution during pregnancy and their contextual determinants in the northeastern region of India
Source: Front Nutr. 2022 Jul 18;9:894245. doi: 10.3389/fnut.2022.894245 (PMC9339897; doi:10.3389/fnut.2022.894245)
Supplement: Supplementary file 2 [file Data_Sheet_2.PDF]

Supplementary Figure 2. Boxplots showing heterogeneities in coverage of micronutrient supplements among districts belonging to different northeastern states. **(A)** Iron-folic acid supplements; **(B)** Calcium supplements. (Coverage indicates number of pregnant mothers who received full course of the supplements per 100 women who registered for antenatal care).

(A)

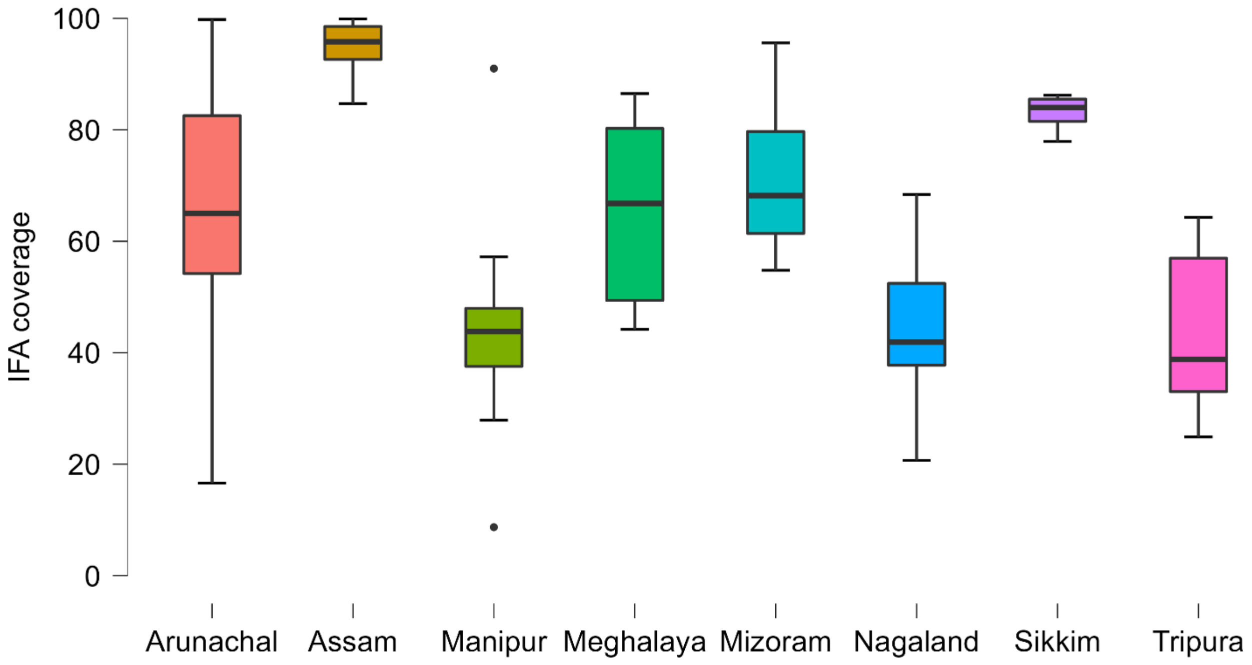

(B)

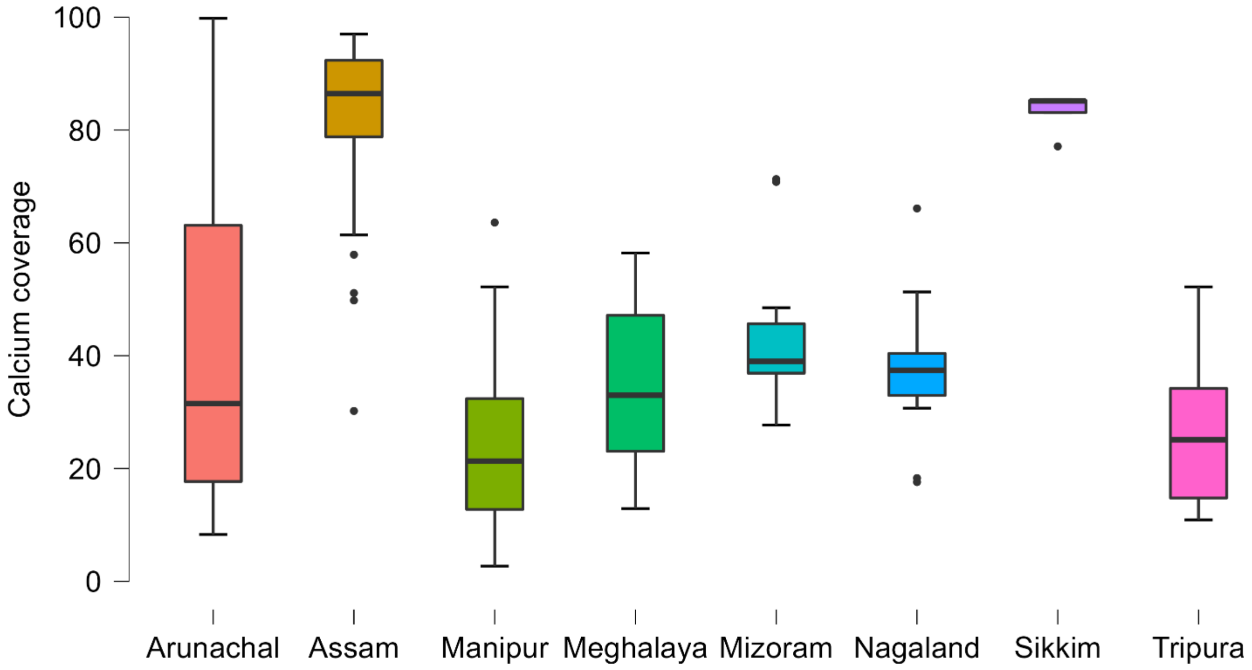
Calcium coverage
100
80
60
40
20
0
Arunachal
Assam
Manipur
Meghalaya
Mizoram
Nagaland
Sikkim
Tripura
