# Supplementary material for: Coverage of antenatal iron-folic acid and calcium distribution during pregnancy and their contextual determinants in the northeastern region of India
Source: Front Nutr. 2022 Jul 18;9:894245. doi: 10.3389/fnut.2022.894245 (PMC9339897; doi:10.3389/fnut.2022.894245)
Supplement: Supplementary file 3 [file Data_Sheet_3.PDF]

Supplementary Table 1. Multivariate regression results showing effect of the explanatory variables on antenatal iron-folic acid (IFA) and calcium coverage.

**Explanatory variables (Contextual factors)**		**Antenatal IFA coverage**				**Antenatal calcium coverage**		
		***β*-coefficient**	***P*-value**	**Model characteristics**		***β*-coefficient**	***P*-value**	**Model characteristics**
Proportion of early ANC visits		0.09	0.59	R^2 = 0.234, adjusted R^2 = 0.202, F-value = 7.47, $P < 0.001$		0.06	0.77	R^2 = 0.220, adjusted R^2 = 0.188, F-value = 6.92, $P < 0.001$
Physiographical category (hilly districts)		-29.92	< 0.001			-27.59	< 0.001	
Physiographical category (plateau districts)		-12.88	0.09			-26.51	< 0.01	
Socioeconomic development category (aspirational districts)		6.71	0.29			3.16	0.68	

Abbreviations: IFA, iron-folic acid; ANC, antenatal care.
