# Supplementary material for: Coverage of antenatal iron-folic acid and calcium distribution during pregnancy and their contextual determinants in the northeastern region of India
Source: Front Nutr. 2022 Jul 18;9:894245. doi: 10.3389/fnut.2022.894245 (PMC9339897; doi:10.3389/fnut.2022.894245)
Supplement: Supplementary file 4 [file Data_Sheet_4.pdf]

Supplementary Table 2. Anemia and pre-eclampsia burden in pregnant women from the states of northeast India, 2018–19 to 2020–21.

**Region State**	**Anemia during pregnancy**†	**Pre-eclampsia**‡
India (national level)	52.2	28.7
Northeastern states		
Arunachal Pradesh	27.9	38.7
Assam	54.2	29.6
Manipur	32.4	29.0
Meghalaya	45.0	42.2
Mizoram	34.0	49.2
Nagaland	22.2	34.3
Sikkim	40.7	41.0
Tripura	61.5	49.4

† The anaemia during pregnancy estimates refer to the prevalence of anaemia (defined as hemoglobin concentration < 11.0 g/dL) detected in pregnant women aged 15–49 years during the National Family Health Survey (NFHS-5) in the 2019–20 period [1]. These estimates are expressed in percentage.

‡ Reliable state-wise estimates of pre-eclampsia prevalence in India are unavailable. The estimates provided in this table are based upon recently published reports on self-reported prevalence of symptoms suggestive of pre-eclampsia during pregnancy among women aged 15–49 years [2,3]. These estimates are expressed per 100 live births.

References:

1. International Institute for Population Sciences (IIPS) and ICF. National Family Health Survey (NFHS-5), India, 2019-21. Mumbai: IIPS (2021).

2. Malik A, Jee B, Gupta SK. Preeclampsia: disease biology and burden, its management strategies with reference to India. Pregnancy Hypertens. (2019) 15:23-31. doi:10.1016/j.preghy.2018.10.011.

3. Agrawal S, Walia GK. Prevalence and risk factors for symptoms suggestive of pre-eclampsia in Indian women. J Womens Health Issue Care. (2014) 3:6. doi: 10.4172/2325-9795.1000169.
